# Supplementary material for: Effects of extreme climate events and child mortality on total fertility rate in Bangladesh
Source: Heliyon. 2024 Jul 23;10(15):e35087. doi: 10.1016/j.heliyon.2024.e35087 (PMC11336454; doi:10.1016/j.heliyon.2024.e35087)
Supplement: Multimedia component 1 [file mmc1.pdf]

Clean Version_HELIYON_31.May.2024.doc

WORD COUNT 11513
TIME SUBMITTED 01-JUL-2024 06:59PM
PAPER ID 109972609

Effects of extreme climate events and child mortality on total fertility rate in Bangladesh

Shah Md Atiqul Haq[1*], Muhammad Abdul Baker Chowdhury[2], Khandaker Jafor Ahmed[3], Md Jamal Uddin[4]

[1]Department of Sociology, Shahjalal University of Science and Technology, Sylhet 3114, Bangladesh. Email: shahatiq-soc@sust.edu; shahatiq1@yahoo.com; https://orcid.org/0000-0001-9121-4028

[2]Department of Neurosurgery, University of Florida, Gainesville, FL 32608, USA. Email: mchow023@fiu.edu; chowdhurym@ufl.edu; https://orcid.org/0000-0002-2145-0938

[3]School for Environment and Sustainability (SEAS), University of Michigan, Dana Building, 440 Church Street, Ann Arbor, MI, 48109. Email: kjafor@umich.edu; https://orcid.org/0000-0003-4409-5710

[4]Department of Statistics, Shahjalal University of Science and Technology, Sylhet 3114, Bangladesh and Department of General Educational and Development, Daffodil International University, Dhaka, Bangladesh. Email: jamal-sta@sust.edu; https://orcid.org/0000-0002-8360-3274

* Corresponding author

Disclosure/Conflict of Interest: The authors declare that they have no potential conflicts (financial, professional or personal) to disclose.

Funding: The authors have received no specific funding from public, commercial or non-profit funding agencies.

Availability of data and material: The results presented in the manuscript, based on secondary data, are available free of charge from EM-DAT, the World Bank and the United Nations.

Code availability: Available on request from the corresponding author.

Author contributions:

Shah Md Atiqul Haq: Literature review, Conceptualization, Data curation, Methodology, Supervision, Original draft preparation, Reviewing, Editing, and Finalizing

Muhammad Abdul Baker Chowdhury: Data curation, Methodology, Reviewing, Finalizing

Khandaker Jafor Ahmed: Literature review, Methodology, Original draft preparation, Reviewing, Editing, and Finalizing

Md Jamal Uddin: Methodology, Reviewing, Editing, and Finalizing

Ethics approval:

The present study was not submitted to an ethics committee for approval, as we used publicly available de-identified data.

Abstract

Floods, storms, and temperature extremes are examples of extreme weather events that have a substantial influence on a country's demographic dynamics, including migration, fertility, and mortality. Changes in population size, composition, and distribution may result from these occurrences. This study, which spans the years 1966 to 2018, looks at how Bangladesh's total fertility rate (TFR) is affected by extreme weather events and child mortality, including neonatal, infant, male infant, and under-five mortality. We use data from secondary publically accessible sources, such as the World Bank and The Emergency Events Database (EM-DAT), and we investigate the correlations using the autoregressive integrated moving average model (ARIMA), complemented by bivariate and multivariable analyses.

Our findings from the univariate analysis are noteworthy. Total extreme climate events (β=-0.345, 95% CI: -0.510, -0.180), as well as individual extreme climate events, such as extreme temperatures (β=-1.176, 95% CI: -1.88, -0.47), floods (β=-0.644, 95% CI: -1.0729, -0.216), and storms (β=-0.351, 95% CI: -0.63159, -0.07154), exhibited negative associations with the TFR. Additionally, factors such as contraceptive prevalence rate (CPR) (β=-0.085, 95% CI: -0.09072, -0.07954) and gross national income (GNI) per capita (β=-0.003, 95% CI: -0.0041123, -0.0024234) were negatively correlated with the TFR. Conversely, various categories of child mortality, namely, infants (β=0.041, 95% CI: 0.040474, 0.042748), males (β=0.038, 95% CI:0.037719, 0.039891), and under-five (β=0.026, 95% CI:0.025684, 0.026979) – are positively associated with TFR. Controlling for two pivotal confounding factors, time and GNI per capita, yielded consistent results in the multivariate analysis. These findings provide insight on the dual impact of extreme weather events, which can reduce TFR while also raising it through infant mortality. This phenomena may be due to the increased vulnerability of younger children in climate-event-prone areas, prompting parents to seek extra children as both a replacement for lost offspring and an insurance mechanism against future child loss.

Keywords: Floods; Storms; Extreme temperature events, child mortality, total fertility rate (TFR), Bangladesh.

1 Introduction and background

Scholars underscore the urgent need to investigate the intricate relationship between disasters, child mortality, and fertility, especially in areas experiencing rapid population growth, such as Bangladesh [1]. Building on this foundation, subsequent studies by Casey et al. and Muttarak emphasize the profound influence of disasters on fertility rates [2,3]. As climate change exacerbates global vulnerability, it is crucial to explore how severe weather events impact fertility rates. Bangladesh has been the subject of numerous fertility studies [4,5,6], examining various trends and shifts.

Recent research in Bangladesh reveals that families who experience damaged homes often seek refuge in temporary storm shelters, where women typically reside while men repair their homes [7,8]. This finding suggests that extreme climate events might increase fertility in highly disaster-prone areas, potentially due to an increased preference for male children [6]. In the aftermath of catastrophic weather events and displacement to temporary shelters, families may feel a need for more male children to assist with labor-intensive chores such as restoring destroyed homes or maintaining household livelihoods. However, a research conducted by Haq and Ahmed in Bangladesh contradicts this premise, indicating that women in areas more severely impacted by floods and cyclones have lower fertility rates than those in regions with fewer extreme climatic events [9]. For example, the devastating floods in Bangladesh in 1988 resulted in illness and mortality, notably among infants under one year old, with drowning emerging as the leading cause of death among children aged 1-4 years [10]. Drowning rates in Bangladesh exceed those in other developing countries, with rural communities, which are socioeconomically vulnerable to catastrophic weather events such as floods and cyclones, suffering the burden [10]. Furthermore, exposure to external risk factors, such as severe weather occurrences, may increase neonatal death rates.

In Bangladesh, several research studies have highlighted the socio-economic factors that influence fertility and the birth choices of sons and daughters. These factors include education, income, poverty, women's empowerment, employment opportunities [11], family planning awareness and societal influence [12]. Fertility rates vary considerably between urban and rural areas, as well as between regions [13]. Disadvantaged socio-economic groups are more vulnerable to violent climatic disasters [1]. Women from lower socioeconomic backgrounds are less likely to want more children when temperatures are high, and are more likely to use family planning methods [14]. Wealthier people often have less desire for children than their peers of lower economic status [15]. In addition, the number of children per woman decreases as monthly household income increases [16]. Floods have a considerable impact on the demographic mobility of poor people living in flood-prone areas [17] and can influence contraceptive use [18].

Recognizing the substantial contributions of previous research elucidating the various factors guiding fertility transitions in Bangladesh, such as education, employment, age at marriage, contraceptive prevalence and socio-cultural aspects such as religion [15,19,20,21,22,23,24-25], our study moves forward. We broaden the horizons of our investigation by looking at the field of disasters and infant mortality, and their influence on fertility rates in Bangladesh.

Bangladesh stands out for the combined efforts of governmental and non-governmental groups in the field of family planning. Major organizations such as World Vision Bangladesh, BRAC Bangladesh and the International Centre for Diarrhoeal Disease Research, Bangladesh (ICDDRB) have worked continuously with the Bangladesh government, forming important partnerships that have shaped health initiatives and provided reproductive health services to a wide range of rural and remote areas [26]. Since its inception in the 1970s, Bangladesh's National Family Planning Program (NFP) has benefited from funding from a number of domestic and foreign donor organizations, advancing research methodologies and facilitating effective program delivery. Family Welfare Assistants (FWAs), or field workers, are an essential part of the outreach services offered to married couples by the Bangladesh Family Planning Department. Every two months, these family assistants stay in touch with the same families or couples, register new marriages, encourage the adoption of family planning, distribute contraceptives and help with referrals to family planning centers for prenatal, postnatal and spontaneous vaginal births [27].

The contraceptive prevalence rate (CPR) in Bangladesh has increased continuously since it was first introduced in 1975. The CPR increased by 54% between 1975 and 2014, and this tendency has persisted. The latest statistics indicates that 62% of married women in Bangladesh who are of reproductive age utilize modern methods of contraception, out of 52% of these women. With 16% of users coming from the social work field and 40% from the public sector, social workers are the primary source of modern contraceptive usage. However, 5% of modern method users receive help from non-governmental organizations [28].

Bangladesh is experiencing an increasing frequency of storms and floods, both exacerbated by the devastating effects of climate change. To understand the complex link between extreme weather events and their effects on fertility, this study drew on historical patterns across a range of disaster categories, including floods, storms, severe cold spells, and mortality inequalities, including neonatal, male and under-five mortality. While many studies have been carried out on how extreme weather conditions affect mortality rates, not as much is known about how catastrophic weather events affect fertility dynamics. The existing literature on this subject presents conflicting results, with some studies suggesting that fertility rates may increase [29,30] while others propose a decline [31,32] following major climatic disasters. In order to better understand the complexities surrounding population policies and the effects of catastrophic

weather events and climate change, this study examined the influence of various forms of extreme weather events, focusing particularly on newborn, male and child mortality, on the total fertility rate (TFR). This study specifically looked at the relationship between several types of extreme weather events and death rates in Bangladesh, a developing country where children are particularly vulnerable to the consequences of these occurrences [33, 34]. This study's primary goal was to investigate the associations between Bangladesh's overall fertility rates, infant mortality, and various types of extreme weather events.

This study examines the complex links between different disasters, indicators of infant mortality and the TFR in Bangladesh, offering a new perspective on the dynamics of climate and fertility. It examines how certain categories of infant mortality, floods and storms interact to affect fertility patterns as a whole. This study highlights the interdependence of these variables rather than their separate assessment, enhancing our understanding of the complex relationship between climate and fertility. Our study aimed to evaluate the impact of various extreme climate events on Bangladesh's TFR and examine the influence of child mortality on TFR. The present study also investigated the role of CPR in this context to contribute to targeted interventions and family planning strategies. These findings contribute to understanding the dynamics between climate and fertility and highlight the importance of addressing child mortality in reproductive health policy and recognizing regional differences and sociocultural factors in developing well-informed social policies.

2 Literature review

The impact of extreme climate events is multifaceted, including various dimensions such as socioeconomic conditions [7, 35]; population dynamics, including both structure and size [1, 6]; reproductive choices [33, 34, 36]; and mortality, specifically neonatal, infant, and child mortality [1, 6, 37, 38]. Severe weather events have direct and indirect impacts on demographic dynamics. These effects include changes in socioeconomic conditions, child mortality, migration and relocation patterns, access to reproductive health services, and the emergence of poor nutritional status [37, 38]. These events also impact the desire to have a son or daughter at birth, as well as fertility [34]. The advantages of having male children who can assist parents in crises and in adverse weather circumstances may be connected to this desire for sons.

Numerous research' empirical findings highlight the complex relationship between extreme weather events like temperature and precipitation and reproduction. For example, in the United States, higher temperatures have been found to cause a nine-month decline in birth rates [45, 46]. Even after adjusting for age and education, Cho revealed how a day with a high temperature of

30-32°C lowers the birth rate in South Korea compared to a day with a temperature of 28–30°C, indicating a decline in pregnancies linked to severe temperatures [47]. Furthermore, in Indonesia, delayed rainy seasons in the previous year were associated with higher fertility and lower use of family planning techniques [14]. Compared to unaffected areas, the consequences of a storm can result in a prolonged interval of 4-6 years before fertility rates return to normal in disaster-affected regions [30]. Flood research, such as studies by Tong et al [48], show variations in birth rates in North Dakota between 1994 and 1996 and between 1997 and 2000. The birth rate fell from 13.1 births per 1,000 inhabitants before the crisis to 12.2 births per 1,000 inhabitants after the crisis. Fertility patterns throughout the post-storm era, however, were contradictory, with some research showing an increase [29, 30] and others showing a decrease [49, 50]. On the other hand, low fertility has continuously been linked to periods of intense heat [14, 46, 47, 51, 52, 53]. Above-average or severe rainfall, particularly in areas like Bangladesh, Indonesia, Mali, Mexico, and sub-Saharan Africa, is associated with higher birth rates than temperature-related occurrences [14, 41, 42, 53, 54].

In addition, there is a strong correlation between mortality and fertility [55]. Notably, people often choose to have more children in response to anticipated disaster risks [9], motivated by the need to replace lost children [56], which in turn influences decisions to have more children [57]. The cases of women who had no children before the 2004 Indian Ocean tsunami and who reported that they planned to become pregnant soon after the event [40]. Couples who have lost one child may decide to try for another to compensate for the one they have lost [58]. On the other hand, some at-risk individuals may be less inclined to desire children following climatic shocks. When extreme weather events become more frequent, people often worry about the increased risk of losing their children [59]. The belief that having more male offspring could contribute to recovery and repair efforts during and after crises [33, 40, 60] and provide a kind of insurance against extreme weather disasters [55, 60] may be behind this trend.

The preference for male offspring in areas vulnerable to severe weather is sometimes attributed to the idea that sons confer advantages. In these areas, male children can provide physical labor, financial support and security during hard times, so families may see them as a kind of insurance or security. Sons are also expected to take care of their parents as they age, acting as a long-term safety net. This view confirms the preference for male offspring, particularly in regions where climate change risks are high.

3 Methodology

3.1 Data source and study population

Secondary data on extreme weather events, mortality and fertility in Bangladesh were analyzed for the study from a variety of open-access sources. The Emergency Events Database (EM-DAT) is a comprehensive archive of extreme weather events dating back to 1900. It can be accessed at https://www.emdat.be/. The International Federation of Red Cross and Red Crescent Societies, government agencies, research groups and insurance companies are among the sources of data collected for this database, which is managed by the Centre for Research on the Epidemiology of Disasters (CRED). For disaster-related research, EM-DAT is widely used by economists, public health researchers and child health researchers [39]. It is important to note that once the user has registered and logged in, EM-DAT is available for free, non-commercial use.

The data provided by EM-DAT for each disaster event include critical details, including the event's location, classification (e.g., severe weather, floods, storms), commencement and conclusion dates, and associated impacts (e.g., fatalities, injuries, displacement, and estimated damages). In the present study, information on disasters in Bangladesh from 1966 to 2018 was extracted using EM-DAT's online data query tool. These recorded events included climatic, hydrological, and meteorological threats, with a particular focus on extreme temperature events, including heat waves (maximum temperature values in °C) and cold waves (minimum temperature values in °C). Additionally, floods and storms were considered as the primary risk categories. The individual variables extracted from the database were obtained annually. We conducted our analysis by summing the number of disaster events annually to understand trends over time.

A cold wave, according to EM-DAT, is characterized by unusually low temperatures that last for two or more days and may be made worse by strong winds. The precise temperature thresholds used to identify a cold wave differ depending on the region. In a similar vein, a heat wave is described by EM-DAT as a stretch of particularly hot and/or humid weather that persists for two or more days. Once more, the exact temperature thresholds used to identify heat waves differ depending on the region. If a heat wave or cold wave is proclaimed by the Bangladesh Meteorological Department (BMD), it is recorded by EM-DAT. To characterize weather patterns, the Bangladesh Meteorological Department uses several classifications for hot and cold waves. They are classed into three types: mild cold snaps (when the minimum temperature is between 8 and 10°C), moderate cold snaps (when the minimum temperature is between 6 and 8°C), and severe cold snaps (when the minimum temperature is less than 6°C). Similarly, heat waves are classed as mild, moderate, or severe based on their peak temperature. A heat wave is classed as

mild if the highest temperature is between 36 and 38°C, moderate if it is between 38 and 40°C, and severe if it is higher than 40°C [61].

Storms in Bangladesh include various types, such as derechos, hail, lightning/thunderstorms, sand/dust storms, storm surges, tornadoes, winter storms/blizzards, extra-tropical storms, and tropical cyclones. Our study broadly considers storms, regardless of their type.

These are the broad flood categories defined by EM-DAT; our study includes all floods regardless of type. Floods include riverine flooding (overflow from stream channels onto dry land), coastal flooding (higher-than-normal coastal water levels), flash floods (rapid flooding from heavy rainfall), and the ponding of water after rainfall. Coastal floods result from tidal changes or thunderstorms and can last for days to weeks, while flash floods occur rapidly due to heavy rainfall and riverine floods result from overflow onto adjacent floodplain land.

The World Bank database, which can be accessed at https://data.worldbank.org/, provided the fertility statistics as well as related variables including TFR, contraceptive prevalence rate, newborn mortality, infant mortality, male infant mortality, and under-five mortality. Notably, detailed household-level data was not included in this analysis, which mostly relied on aggregated data at the national level. Although household-level data would have yielded more complex insights and strengthened the finding, they were outside the purview of this study. In addition, the research did not include monthly data due to the very low frequency of climate-related disasters for Bangladesh. In particular, the monsoon season is when extreme weather phenomena such as floods, storms and sharp temperature variations are most frequent. Future studies could, however, combine monthly demographic data with disaster data to examine migration, mortality, fertility and the direct and indirect effects of seasonal changes on these variables.

Finally, it should be mentioned that this study was exempted from ethical review because it uses exclusively anonymous data that are readily available to the public.

3.2 Outcome variable

The TFR was the main outcome variable in this study. The total fertility rate (TFR) is a measure of the average number of children a woman would have if she lived to the end of her reproductive potential. Based on data from the United Nations Population Division, the World Bank has methodically collected total fertility rate data for Bangladesh.

3.3 Predictors

Several predictors were considered in this analysis. The annual counts of [deaths, injuries, affected individuals, and homelessness], as well as the total number of people impacted by these catastrophes, were among the variables associated to disasters. The study also examined characteristics associated to climate events, such as the overall number of extreme climatic events that occurred between 1966 and 2018. Additionally, a more thorough analysis of specific records pertaining to floods, storms, and extremely high temperatures was carried out. Flood types were further divided into subcategories, such as river, coastal, and flood occurrences, under these categories. Tropical cyclones and convective storms are two of the storm subtypes. Severe winter conditions, heat waves, and cold waves are characteristics of extreme temperature occurrences. After a thorough evaluation of the literature, the fertility predictors were selected with care, taking into account insights from the fields of public health, sociology, anthropology, and demography.

3.4 Model construction

The total number of extreme climatic events was the key predictor used in the current study's five different models to anticipate the outcome variable TFR. To offer a thorough study, further predictors were subsequently included to each model. While newborn mortality was included in the second model, under-five mortality (male and female) was included in the first. Infant mortality was taken into account in the third model, whereas male infant mortality was linked to the fourth model. CPR and gross national income (GNI) per capita were included as the elements under investigation in the fifth and final model. Notably, in this study, GNI and CPR were thought to be potential control factors.

As earlier research [62,63] have shown, it is important to emphasize that CPR substantially effects fertility drop in low-income, lower-middle-income, and upper-middle-income nations, holding all other factors constant. Moreover, studies have shown that the overall fertility rate of a nation is less affected by economic metrics like GDP per capita [41, 64]. Notably, Chen et al. found that Bangladesh's overall fertility and infant mortality rates were significantly impacted negatively by GDP per capita. Furthermore, they found a responsive link in both the short and long periods between GDP per capita and Bangladesh's real fertility rate [41].

While GDP and GNI are important indicators of economic progress, GNI is clearly superior than GDP when taking Bangladesh's economic environment into account. This benefit results from the GNI's inclusiveness, which accounts for overseas outlays and receivables. All sources of revenue, both foreign and local, that enter the national economy are fundamentally included in the gross

national product (GNI). Remittances are Bangladesh's second-largest external source of foreign revenues after exports, and they are crucial to the country's economic growth and development [65,66]. Remittances into Bangladesh have increased significantly, according to the Bureau of Manpower, Employment, and Training [BMET]; they went from USD 1.95 billion in 2000 to an astounding USD 22.07 billion in 2021 [67].

Individual extreme weather events (such as extreme temperatures, floods and storms) were added to each of the five models for further examination. This was done in order to compare variances with the main model, which uses the number of extreme weather events as the main predictor variable.

3.5 Equations

We first conducted univariate regression analyses to understand the individual impacts of various predictors on the TFR. The regression equation used for this analysis was as follows:

$$TFR = \beta_0 + \beta_1 X_1 + \epsilon$$

Where:

- TFR represents the total fertility rate,
- X_1 denotes the predictors such as total extreme climate events/extreme temperature events/ floods/storms/under-five mortality/neonatal mortality/infant mortality/male infant mortality/contraceptive prevalence rate/GNI per capita,
- β_0, β_1 are the regression coefficients, and
- ϵ is the error term.

These coefficients provide insights into the direction and magnitude of the association between each predictor and the total fertility rate.

We then investigated the relationship between the TFR and the total number of extreme climate events, along with other characteristics. The regression equation for this analysis is as follows:

$$TFR = \beta_0 + \beta_1 X_1 + \beta_2 X_2 + \beta_3 X_3 + \epsilon$$

Here, X_1 represents the total extreme climate events, X_2 denotes the under-five mortality rate/ neonatal mortality rate/infant mortality rate/male infant mortality rate, and X_3 corresponds to the

GNI per capita. The coefficients $\beta_0, \beta_1, \beta_2, \beta_3$ estimate the impact of each predictor on the TFR, while ϵ signifies the error term.

Additionally, we extended our analysis to include time as a predictor variable to capture any temporal trends in the relationship between the TFR and the total number of extreme climate events, along with other characteristics. The regression equation for this model is as follows:

$$TFR_t = \beta_0 + \beta_1 X_1 + \beta_2 X_2 + \beta_3 X_3 + \beta_4 X_4 + \epsilon t$$

In this equation, TFR_t represents the TFR at time t, X_1 denotes the total extreme climate events, X_2 corresponds to the under-five mortality rate/neonatal mortality rate/infant mortality rate/male infant mortality rate, X_3 represents the GNI per capita, and X_4 signifies time. The coefficients β_0, β_1, β_2, β_3, β_4 estimate the effect of each predictor on the TFR, while ϵt denotes the error term associated with time t.

Moreover, we examined the relationship between the TFR and individual disaster types, along with other characteristics. The regression equation for this model is expressed as

$$TFR = \beta_0 + \beta_1 X_1 + \beta_2 X_2 + \beta_3 X_3 + \beta_4 X_4 + \beta_5 X_5 + \epsilon$$

Here, X_1 represents extreme temperature events, X_2 corresponds to floods, X_3 represents storms, X_4 signifies the under-five mortality rate/neonatal mortality rate/infant mortality rate/male infant mortality rate, and X_5 denotes the GNI per capita. The coefficients $\beta_0, \beta_1, \beta_2, \beta_3, \beta_4, \beta_5$, estimate the impact of each predictor on the TFR, while ϵ represents the error term.

Lastly, we employed ARIMA regression to assess the relationship between the TFR and total extreme climate events alongside other characteristics. The ARIMA regression equation for this analysis is as follows:

$$TFR = \beta_0 + \beta_1 X_1 + \beta_2 X_2 + \ldots + \beta_n X_n + \epsilon$$

Here, $X_1, X_2, \ldots, X_n$ represent predictors such as total extreme climate events, under-five mortality rate, neonatal mortality rate, infant mortality rate, male infant mortality rate, and contraceptive prevalence rate. The coefficients $\beta_0, \beta_1, \beta_2, \ldots \beta_n$, estimate the effect of each predictor on the TFR, while ϵ denotes the error term.

3.6 Statistical analysis

For continuous variables, the analysis of this study included descriptive statistics such as mean, standard deviation, and interquartile range. For categorical data, frequencies and percentages were calculated, and chi-square and/or Fisher's exact tests were used to evaluate connections between categorical variables.

We used both bivariate and multivariate linear regression models to find the major determinants of TFR. Initially, bivariate regression models were used to analyze each predictor's results. The variance inflation factor (VIF) was used to assess the models' degree of collinearity, with a maximum cutoff value of 10. According to other studies [68], only predictors with VIF values less than 10 were kept, enabling further regression analysis. The Akaike information criterion (AIC), the Bayesian information criterion (BIC), and modified R-squared were used to evaluate the performance of the model. The models with the lowest AIC and BIC values and the greatest R-squared values were deemed to be the best ones.

Numerous extreme weather events, different types of extreme weather events, neonatal mortality, infant mortality, male child mortality, under-five child mortality, CPR and GNI were among the factors examined in this study. Above all, this study assessed the annual number of extreme weather events impacting the country as a whole, using time-series data rather than focusing on a particular calamity or year.

The total fertility rate (TFR) in Bangladesh was compared to a number of independent variables, such as child mortality rates, socioeconomic indicators, extreme weather occurrences, and other features, using multivariate regression analysis. With this method, the impacts of several predictors on the end variable TFR may be examined simultaneously, leading to a thorough knowledge of the variables affecting fertility dynamics. We can evaluate each variable's distinct contribution while accounting for the influence of other factors by incorporating multiple predictors into the study. GNI per capita was taken into account consistently throughout these studies because of its importance in comprehending the dynamics of fertility. Keeping other predictors constant, we added this variable to each model to provide light on how total extreme climatic occurrences interact with other factors. Additionally, we examined the unique consequences of other categories of death, such as mortality for boys and girls under the age of five, neonatal mortality, infant mortality, and male infant mortality.

In addition to regression models, an autoregressive integrated moving average (ARIMA) model was used to investigate the relationship between outcome and predictor variables. This model requires historical time-series data of the underlying variables and involves three parameters: p

and q, representing the orders of the autoregressive (AR) and moving average (MA) respectively, and d, indicating the number of general differentiations. The "I" component of the ARIMA model, which stands for "integrated", represents the number of differentiation operations required to achieve stationarity in the time series data. Stationarity is crucial, as it implies that the statistical properties of the data, such as mean and variance, remain consistent over time. As a general rule, stationarity involves determining the differences between consecutive data points. Selection of the best ARIMA model, characterized by the appropriate order of the autoregressive term (p) and the moving average term (q), was determined using the Bayesian Information Criterion (BIC) from among several provisional ARIMA models. The study identified ARIMA (1,1,3) as the optimal model for the data set, as it had the lowest BIC value (-415.20). The same predictors and models used in the linear regression were employed, and parameter estimation was carried out using the maximum likelihood method.

The distinct insights provided by ARIMA models are highlighted by contrasting the findings of ARIMA regression with those of other regression models, such as multivariate regression. ARIMA models offer more information about the temporal patterns and trends in the data than multivariate regression models, which only evaluate the relationship between extreme climatic events and TFR.

The multivariate regression models' estimations of the regression coefficients, standard errors, p-values, and 95% confidence intervals (CIs) were also provided. Furthermore, the models' multicollinearity was evaluated by the use of the variance inflation factor (VIF). Variables that exceeded the widely recognized threshold for multicollinearity, which is a VIF value of 5, were eliminated from the model. STATA 15.0 (StataCorp LP, College Station, Texas, USA) was used for the statistical analyses. A two-sided test was used for all tests, and a p<0.05 threshold was selected for statistical significance.

4 Results

4.1 Trends in dependent and independent variables

Between 1966 and 2018, Bangladesh encountered a series of significant historical extreme climate events including various climate hazards. **Table 1** provides a comprehensive overview of these events from 1900 to 2018. Notably, floods and storms have emerged as the predominant types of extreme climate events, recurrently affecting the nation. In particular, river floods and tropical cyclones have been more prevalent than other climate-related occurrences, impacting numerous residents and resulting in substantial damage. **Figure 1** illustrates that over the past decade, the number of extreme climate events decreased in comparison to the preceding two

decades, spanning from 1990 to 2010. During the past three decades, Bangladesh has experienced over ten extreme climate events.

Figure 2 offers insights into the fertility trends in Bangladesh from 1966 to 2018. Notably, the TFR exhibited a remarkable decline from seven children per woman in 1966 to a replacement level of 2.1 children per woman in 2018. From 2000 to 2018, the country successfully reduced its total fertility rate by one child per woman. Furthermore, Figure 2 highlights the substantial growth in Bangladesh's CPR, which rose from nearly zero in the 1980s to approximately 50% in the late 2000s. Remarkably, CPR remained consistently between 50% and 60% for almost two decades, before surpassing 60% in 2015. The trends in TFR and CPR align, indicating that a decline in TFR correlates with an increase in CPR.

Infant and male infant mortality rates decreased to fewer than 50 per 1,000 live births in 2005, and they stayed below 25 per 1,000 live births in 2018, as **Figure 3** illustrates. In addition, in 2018 the rates of neonatal and under-five deaths (for male and female) decreased to about 25 per 1,000 live births. Moreover, compared to other mortality categories, neonatal mortality decreased more rapidly.

Figure 4 provides a comprehensive record of Bangladesh's GNI dating back to 1973, when it amounted to US$ 120. Subsequently, GNI steadily increased until 2000, followed by a dramatic surge, reaching US$ 1,750 in 2018. This upward trajectory in GNI signifies the country's socioeconomic development, which is a factor associated with declining fertility rates.

Furthermore, similar to the linear regression models, the time-series models yielded congruent results regarding parameter estimates and the significance of the covariates. **Table 7** shows identical outcomes concerning the total number of extreme climate events and TFR in Models 1, 3, and 4, all bearing statistical significance at 0.05. Furthermore, across all models, the findings consistently revealed an inverse relationship between extreme climate events and fertility, implying that more extreme climate events correlate with decreased fertility rates. Pertaining to mortality differentials, the ARIMA analysis likewise underscores the contribution of under-five mortality and neonatal mortality to increased fertility, demonstrating a significance level of 0.01. Detailed results are presented in **Table 7**.

4.2 Descriptive statistics of selected variables

Table 2 provides descriptive statistics for the selected variables and offers insights into key indicators. On average, Bangladesh experienced approximately 5.26 (SD=2.68, range 0-12)

extreme climate events annually, reflecting the country's susceptibility to such occurrences. CPR exhibited an average of 45.67% (SD=16.01, range 7.7-62.4), indicating the proportion of women currently using contraceptives.

The average TFR stood at 4.42 (SD=1.83, range 2.04-6.95). Notably, Bangladesh witnessed its highest TFR (6.95 between 1969 and 1970, whereas the lowest was recorded at 2.04 in 2018. This dynamic range underscores the significant changes in fertility patterns over the years.

Examining mortality rates, the average neonatal mortality rate was 58.92 (SD=27.04, range 17.1-94.9) per 1000 live births, while the rate for children under five years of age averaged at 131.01 (SD=69.37, range 30.2-230.2) per 1000 live births. Furthermore, the average infant mortality rate stood at 89.63 (SD=44.35, range 25.6-153.3) per 1000 live births, and the average male infant mortality rate was 95.84 (SD=47.55, range 27.3-164.6) per 1000 live births.

Finally, considering the GNI per capita, the average was US$ 508.70. Notably, GNI has experienced recent growth, with Bangladesh transitioning to the status of a lower-middle-income country. These statistics collectively provide a comprehensive overview of the factors under examination, illuminating the country's demographic and economic landscapes.

4.3 Univariate effect: predictors and TFR

The regression coefficients of the factors predicting changes in TFR are shown in **Table 3**. The findings show that there is substantial explanatory power for variability in TFR across all factors. We looked at the overall number of extreme climatic occurrences and particular catastrophe aspects individually to understand their respective effects on the TFR.

The overall number of extreme climatic events and TFR showed a substantial inverse relationship. When certain extreme climatic events are taken into account, this detrimental influence becomes more evident. When comparing these to other specific extreme climatic occurrences and chosen predictors, extreme temperature showed the largest coefficient. In particular, there was a significant drop in average fertility of 1.17 units for every unit increase in severe temperature occurrences.

Nonetheless, TFR was positively and significantly impacted by a number of infant mortality factors. Neonatal mortality had the strongest effect of all these parameters. For example, there was a 0.067 unit increase in fertility for every unit rise in newborn death. In a similar vein, TFR increased by 0.03, 0.04, and 0.04, respectively, in relation to under-five mortality for both genders, infant mortality, and male infant mortality.

Additionally, this study showed that a 0.09 unit (P<.05) decrease in TFR was linked to a one unit increase in CPR. Put another way, average fertility fell by 0.003 units for every unit increase in GNI per capita.

4.4 Multivariable effects: predictors and TFR

Table 4 illustrates the positive link between the TFR and all types of mortality based on the findings of these regression models. Interestingly, compared to Models 1, 3, and 4, newborn mortality in Model 2 had a greater coefficient than the other categories of death. Next, we ran a separate model (Model 5) that included GNI and CPR in addition to the number of extreme weather events in order to estimate their impact on the TFR. Surprisingly, the results show that, at $p < 0.10$, only extreme weather events had a meaningful impact on fertility rates.

In **Table 5**, we present the results of multivariate regression models examining the relationship between TFR and various predictor variables. The total number of extreme climate events demonstrates a consistent negative association with TFR across the models, although with varying levels of statistical significance. For instance, in Model-1, the coefficient for extreme climate events is -0.0210 ($p = 0.025$), indicating that an increase in extreme climate events is associated with a decrease in TFR. This negative association persists in Models 3 and 4, with coefficients of -0.0290 ($p = 0.005$) and -0.0314 ($p = 0.003$), respectively.

Mortality rates also play a significant role in shaping fertility patterns. For example, in Model-2, the coefficient for neonatal mortality rate is 0.0790 ($p < 0.001$), suggesting that areas with higher neonatal mortality rates tend to exhibit higher fertility rates. Similarly, in Model-4, the coefficient for male infant mortality rate is 0.0232 ($p = 0.015$), indicating a positive association between male infant mortality and TFR.

Moreover, socioeconomic factors contribute to variations in TFR. Across all models, an increase in gross national income (GNI) per capita is consistently associated with higher TFR. For instance, in Model-3, the coefficient for GNI per capita is 0.0007 ($p = 0.001$), suggesting that higher GNI per capita is linked to higher fertility rates. Conversely, contraceptive prevalence rate exhibits a negative association with TFR. In Model-5, the coefficient for contraceptive prevalence rate is -0.0311 ($p < 0.001$), indicating that higher contraceptive prevalence rates are associated with lower fertility rates.

The inclusion of time as a predictor variable allows for an assessment of temporal trends in TFR over the study period. While the coefficients for time vary across models, indicating non-

significant associations in some instances, Model-5 reveals a significant negative relationship between time and TFR (β = -0.1018, $p < 0.001$) when contraceptive prevalence rate and GNI per capita are considered. This finding suggests a notable decline in TFR over time, particularly in areas with higher contraceptive prevalence rates. By incorporating time into the analysis, policymakers and stakeholders gain valuable insights into the evolving fertility patterns in Bangladesh, facilitating the development of targeted interventions to address changing demographic dynamics and reproductive health needs.

Table 6 provides a range of regression models that examine the impact of several extreme climatic events, including storms, floods, and extreme temperature events, in addition to other factors linked to TFR and child mortality. With the exception of model 5, the majority of models show negative correlations between TFR and floods and severe temperature occurrences. This shows that lower fertility rates are typically found in locations where catastrophic calamities occur more frequently. Specifically, the coefficients for extreme temperature events range from -0.055 to -0.068, while those for floods range from -0.042 to -0.052. These associations are statistically significant ($p<0.05$), indicating a robust relationship between climate-related disasters and fertility behaviors. In contrast, the connection between storms and TFR appears to be modest and statistically insignificant across models. This shows that storms may have a less substantial influence on birth rates in Bangladesh than high temperature events and floods.

Regarding mortality rates, the under-five mortality rate shows a positive association with TFR, with coefficients around 0.027. This association is highly statistically significant ($p < 0.001$), suggesting that regions with higher child mortality rates tend to have higher fertility rates. This underscores the pivotal role of child survival in shaping fertility decisions. Similarly, neonatal and infant mortality rates display positive associations with TFR in models 2 and 3, with coefficients ranging from 0.073 to 0.043. These associations are statistically significant ($p < 0.001$), emphasizing the influence of early childhood mortality on fertility behaviors. Additionally, the male infant mortality rate demonstrates a positive association with TFR in model 4, with coefficients around 0.041. This association is statistically significant ($p < 0.001$), indicating that regions with higher male infant mortality rates may also have higher fertility rates. Interestingly, Model 5 charted a course distinct from the preceding four models. In this context, each unique disaster type displayed a positive relationship with the TFR when CPR was included. Nonetheless, the impacts of extreme temperatures and floods failed to reach statistical significance. Notably, this model posits that fertility increases alongside a rising number of storms but decreases in tandem with increasing CPR.

The ARIMA regression results shown in **Table 7** reveal useful insights into the association between extreme climatic events and total fertility rate (TFR) throughout time, with more subtle

implications than previous regression models. Several major discoveries emerge from an analysis of the ARIMA models' coefficients. To begin, the coefficients represent the magnitude of the influence of extreme climatic events on TFR, taking into account autocorrelation and seasonality in the dataset. For instance, in Model-1, the coefficient for total extreme climate events (-0.0000789) suggests a significant negative association with TFR, implying that an increase in extreme climate events is associated with a decrease in TFR over time (t = -2.15, p = 0.031).

Moreover, ARIMA regression models capture the temporal dynamics of the variables, providing insights into lagged effects and time-dependent patterns. This temporal perspective is particularly evident in Model-3, where the coefficient for total extreme climate events (-0.0000805) remains significant, indicating a negative relationship with TFR even after accounting for the lagged effects of extreme climate events (t = -2.57, p = 0.01). This implies that variations in extreme weather events have a long-term effect on fertility rates, emphasizing how crucial it is to take temporal dynamics into account when figuring out how climate change and reproductive health outcomes are related.

5 Concluding discussions

This study looks at how Bangladesh TFR is affected by different calamities and child mortality. Our study clarifies the relationship between changes in fertility and several forms of child mortality, such as neonatal, infant, male infant, and under-five mortality, as well as widespread extreme climatic events like floods and storms. Notably, throughout the previous two to three decades, TFR trends in Bangladesh have shown a notable drop at the same time that CPR has significantly increased. In addition, there has been a decline in the frequency of several child mortality categories, including neonatal, newborn, male baby, and under-five death.

Statistical analysis revealed significant associations between the total count of extreme climate events, including floods and storms, and TFR, all pointing in a negative direction. In simpler terms, an upsurge in floods and storms is likely to decrease the total fertility rate, corroborating the findings from Lin’s study following tsunamis in Japan, where fertility declined after a disaster event [69]. Similarly, this aligns with the findings of Tong et al. in the United States after a flood event [48].

We observed a noteworthy decrease in fertility rates, particularly in response to increasing flood events, compared with storms in Bangladesh. This can be attributed to the higher frequency and wider geographical coverage of floods in Bangladesh compared with other extreme climate events [65]. In alignment with this effect of floods on fertility, Tong et al. noted a decline in

fertility following a flood event in the United States [48]. Other extreme climatic events, such as Eritrea [70] and Tajikistan [71]), also exhibited a decline in fertility.

According to our research, Bangladesh's overall fertility rate tends to increase when considering all categories of child mortality indicators, including neonatal, infant, male, and under-five. Of particular relevance is the elevated coefficient linked to neonatal death, suggesting that individuals could choose to have a larger family size, potentially due to worries about infant mortality [9]. The likelihood of child mortality will likely increase with an increase in the frequency, intensity, and impact of extreme climatic events. The prospect of losing a child and the approaching risk of such events may encourage parents to think of having additional kids as insurance, a phenomena that has been extensively studied in the past [37, 38, 56].

In addition, we studied the impact of CPR on fertility rates and found a similar trend towards lower TFR with higher CPR. Interestingly, the strength of this effect changes with the number and type of extreme weather events. Interestingly, distinct results were obtained for each calamity with the inclusion of CPR in the model. A statistically significant correlation was found between TFR and the overall frequency of extreme weather events, floods, storms and the different types of child mortality. However, the addition of CPR to Model 5 generated an intriguing dynamic. The coefficients for the interaction between individual extreme climate events and TFR were positive, indicating that TFR rose with more overall extreme climate events but reduced as CPR increased. These findings show that the availability and accessibility of contraception during crises may lead to lower fertility rates, a pattern corroborated by research in affluent nations [18, 72, 73].

Interestingly, during the course of the previous three Demographic and Health Surveys that were carried out in Bangladesh in 2011–2014 and 2017–18, both CPR and TFR showed a steady trend [74]. Furthermore, there are notable differences between districts and administrative divisions in terms of child mortality, overall fertility, and contraceptive usage in Bangladesh [28]. These variations are intrinsically linked to the diverse climatic disturbances experienced by each region, which are influenced by demographic, economic, social, and cultural factors [12,75]. Sociocultural variables, including education, contraceptive utilization, preferences of son or daughter at birth, societal pressures, and religion, have impacted women's preferences for having more children in flood-affected areas [6,9]. According to Haq and Haq and Ahmed, women residing in flood- and cyclone-prone areas tend to have more children overall and aspire to have more children in the future [6,9]. Furthermore, religious beliefs have a bearing on the preference for higher fertility, with non-Muslims expressing a stronger inclination towards larger families than their Muslim counterparts [9]. Consequently, prioritizing disaster risk reduction and the expansion of family planning services in disaster-prone and hard-to-reach areas should be of

paramount concern, as they can contribute to reducing fertility and child mortality rates while improving access to and the use of contraceptives [76].

6 Limitations and future studies

Our study is not without limitations, and it is important to acknowledge that firm conclusions cannot be drawn definitively based solely on the results of this investigation. Several limitations should be considered, primarily associated with the data and the analytical methods.

First of all, it is essential to recognize that fertility is often the result of past choices and actions that may have taken place before the awareness and effects of a calamity. Due to the occurrence of numerous disasters in a single year, we encountered the problem in our study of the impossibility of isolating the impacts of each individual disaster on the total fertility rate at any given time. To provide a more detailed assessment of the effects of individual disasters, future studies could examine fertility before and after a disaster. To do this, scientists should focus on single disasters and use methods such as difference-in-difference analysis to better understand alterations. Although the present study did not undertake such an analysis, elucidating the underlying relationship between extreme weather events, mortality and fertility through cross-national comparisons may provide a more comprehensive understanding and have wider implications in an era marked by rapid climate change and an increase in extreme weather events. In addition, conducting detailed qualitative studies in various disaster-prone areas in developing countries, including Bangladesh, could enhance our understanding of the link between extreme weather events, mortality and fertility in regions characterized by climatic uncertainties. To provide a full picture of fertility behavior, future research may expand their analytical reach by examining multi-temporal patterns, combining new drivers and data sources, such as high-resolution fertility and catastrophe maps, and including additional nations.

The second constraint is the lack of completely comparable time-series data at the village, union, district, and sub-district levels. We used national-level data for our research, and we did not take into consideration differences in the effects of disasters at the district or household levels. District-level data should make it easier to identify places with high and low fertility in relation to catastrophe hotspots, which is an essential direction for future study, especially in areas vulnerable to catastrophic climatic events. Notably, a disproportionate amount of extreme weather occurrences are experienced by some parts of Bangladesh, especially those around the shore. It is possible that the models used in this study failed to account for regional differences in infant mortality, fertility, and the impact of extreme climatic events, underestimating their significance in these coastal regions. Certain locations are probably more impacted by extreme

climate events than others, while other places may be less influenced by a particular type of extreme climatic event than others. With a population of over 170 million, Bangladesh is a diversified country with a range of livelihood practices that might affect fertility dynamics. Therefore, the main focus of future study should be on examining regional differences and subtleties.

Third, while our analysis provides valuable insights into the relationships between extreme climate events, child mortality indicators, and TFR, we conducted annual analyses because of data availability constraints, thereby potentially overlooking the variability at monthly or seasonal levels. Future research should explore these relationships at finer temporal resolution. Fourth, our study did not explicitly consider confounders, such as air pollution and national climate mitigation and adaptation policies, which could influence the observed relationships. Considering these confounders could provide a more comprehensive understanding of climate-fertility dynamics.

One notable limitation inherent in our study is the intricate role of religious factors in shaping fertility decisions. While our research comprehensively investigates the relationships among disaster events, child mortality, and fertility rates in Bangladesh, it does not directly incorporate the impact of religion on these dynamics. Religion undoubtedly plays a substantial role in shaping the attitudes of individuals and communities towards family planning and fertility preferences. However, quantifying this influence is challenging because of the multifaceted nature of religious beliefs and practices. We acknowledge the significance of religion in this context, and future research could delve deeper into the intricate interplay between disasters, religious beliefs, and fertility choices. This exploration could be undertaken through localized studies, providing a more nuanced understanding of how religion influences perceptions of child mortality and individuals' propensity to have more children in the face of disaster risks. Such investigations would contribute to a more comprehensive assessment of climate-fertility dynamics in Bangladesh.

Tables

Table 1 Disaster records for Bangladesh, 1900–2018

**Disaster type**	**Disaster sub-type**	**Count of events**	**Total deaths**	**Population affected**	**Total damage (USD x 1000)**
Extreme temperature	Cold wave	19	2,182	313,200	-
	Heatwave	2	62	-	-
	Severe winter conditions	2	230	101,000	-
Floods	Not specified	35	45,026	185,490,392	4524100
	Coastal flood	2	51	473,335	-

	Flash flood	11	261	7,634,577	729000
	Riverine flood	46	7278	138,644,785	7763300
Storms	Not specified	49	5,706	2,356,857	850,000
	Convective storm	39	2,153	1,470,091	40,401
	Tropical cyclone	89	626,943	82,168,734	5,405,979

Data source: [77]

Table 2 Sample characteristics of selected variables*

Variables	N	Mean	Median	SD	Minimum	Maximum
Total extreme climate events	54	5.26	5	2.68	0	12
Total fertility rate	52	4.42	4.07	1.83	2.04	6.95
Contraceptive prevalence rate	31	45.67	52.64	16.01	7.7	62.4
Neonatal mortality	52	58.92	58.2	27.04	17.1	94.9
Under-five mortality	52	131.01	128.7	69.37	30.2	230.2
Infant mortality	53	89.63	88.4	44.35	25.6	153.3
Male infant mortality rate	53	95.84	94.7	47.55	27.3	164.6

Note: The units of all the variables are annual.

Table 3 Univariate regression coefficient predicting TFR

Variables	β coefficient	Std. Err.	t	p-value	95 % CI	
Total extreme climate events	-0.345484	0.08225	-4.2	<0.001	-0.51069	-0.18028
Extreme temperature events	-1.176515	0.350055	-3.36	0.001	-1.87962	-0.47341
Floods	-0.644639	0.213258	-3.02	0.004	-1.07298	-0.2163
Storms	-0.351564	0.139415	-2.52	0.015	-0.63159	-0.07154
Under-five mortality, both sex	0.026332	0.000322	81.67	<0.001	0.025684	0.026979
Contraceptive prevalence rate	-0.085133	0.002733	-31.15	<0.001	-0.09072	-0.07954
Neonatal mortality	0.067474	0.000943	71.58	<0.001	0.065581	0.069367
Infant mortality rate	0.041611	0.000566	73.5	<0.001	0.040474	0.042748
Male infant mortality rate	0.038805	0.000541	71.76	<0.001	0.037719	0.039891
GNI per capita	-0.0032679	0.0004187	-7.8	<0.001	-0.0041123	-0.0024234

Table 4 Regression of total fertility rate by the total number of extreme climate events and other characteristics

Variables	β coefficient	Std. Err.	t	p-value	95% CI	
**Model-1**						
Total extreme climate events	-0.0199566	0.0082354	-2.42	0.02	-0.036588	-0.003325
Under-five mortality rate, both sex	0.0280016	0.0005654	49.52	<0.001	0.0268598	0.0291435
GNI per capita	0.0004132	0.0000883	4.68	<0.001	0.0002348	0.0005916
**Model-2**						
Total extreme climate events	-0.0131565	0.0065586	-2.01	0.051	-0.026402	0.0000889
Neonatal mortality rate	0.0740423	0.0011764	62.94	<0.001	0.0716665	0.076418
GNI per capita	0.0006461	0.0000728	8.87	<0.001	0.000499	0.0007931
**Model-3**						
Total extreme climate events	-0.0240292	0.0095802	-2.51	0.016	-0.043377	-0.0046816

Infant mortality rate	0.0440981	0.0010442	42.23	<0.001	0.0419893	0.0462068
GNI per capita	0.0004165	0.0001036	4.02	<0.001	0.0002073	0.0006257
**Model-4**						
Total extreme climate events	-0.0259093	0.0099781	-2.6	0.013	-0.04606	-0.0057582
Male infant mortality rate	0.0411862	0.0010192	40.41	<0.001	0.0391278	0.0432446
GNI per capita	0.0004126	0.0001082	3.82	<0.001	0.0001942	0.000631
**Model-5**						
Total extreme climate events	0.0358615	0.0183623	1.95	0.061	-0.001815	0.0735378
Contraceptive prevalence rate	-0.0831272	0.0041195	-20.2	0	-0.09158	-0.0746747
GNI per capita	-0.0003307	0.000206	-1.61	0.12	-0.000753	0.0000919

Table 5 Regression of total fertility rate by the total number of extreme climate events and other characteristics with time added as a predictor

	β coefficient	Std. Err.	t	p-value	95% CI	
**Model-1**						
Total extreme climate events	-0.0210	0.00900	-2.34	0.025	-0.0392	-0.0028
Under five (U5) mortality rate both sex	0.0263	0.00546	4.82	<0.001	0.0153	0.0373
GNI per capita	0.0005	0.00019	2.48	0.018	0.0001	0.0008
Time	-0.0093	0.02980	-0.31	0.756	-0.0696	0.0509
**Model-2**						
Total extreme climate events	-0.0116	0.00733	-1.58	0.122	-0.0264	0.0032
Neonatal mortality rate	0.0790	0.01005	7.86	<0.001	0.0587	0.0994
GNI per capita	0.0006	0.00011	5.32	<0.001	0.0004	0.0008
Time	0.0104	0.02081	0.5	0.619	-0.0316	0.0525
**Model-3**						
Total extreme climate events	-0.0290	0.00982	-2.96	0.005	-0.0489	-0.0092
Infant mortality rate	0.0285	0.00925	3.08	0.004	0.0098	0.0472
GNI per capita	0.0007	0.00020	3.53	0.001	0.0003	0.0011
Time	-0.0543	0.03197	-1.7	0.097	-0.1190	0.0103
**Model-4**						
Total extreme climate events	-0.0314	0.01004	-3.13	0.003	-0.0517	-0.0111
Male infant mortality rate	0.0232	0.00918	2.53	0.015	0.0047	0.0418
GNI per capita	0.0008	0.00021	3.65	0.001	0.0003	0.0012
Time	-0.0667	0.03392	-1.97	0.056	-0.1352	0.0019
**Model-5**						
Total extreme climate events	-0.0037	0.00868	-0.42	0.676	-0.0215	0.0142
Contraceptive prevalence rate	-0.0311	0.00507	-6.12	<0.001	-0.0415	-0.0206
GNI per capita	0.0009	0.00014	6.25	<0.001	0.0006	0.0012
Time	-0.1018	0.0093	-10.95	<0.001	-0.1209	-0.0827

Table 6 Regression of total fertility rate by individual disaster and other characteristics

Variables	β coefficient	Std. Err.	t	p-value	95% CI	
Model-1						
Extreme temperature events	-0.0552378	0.0259036	-2.13	0.039	-0.1076329	-0.0028428
Floods	-0.0429487	0.0170406	-2.52	0.016	-0.0774167	-0.0084808
Storms	-0.0053884	0.011295	-0.48	0.636	-0.0282347	0.0174579
Under-five mortality rate, both sex	0.0277655	0.000566	49.05	<0.001	0.0266206	0.0289104
GNI per capita	0.0003888	0.0000876	4.44	<0.001	0.0002115	0.000566
**Model-2**						
Extreme temperature events	-0.0491295	0.0204375	-2.4	0.021	-0.0904682	-0.0077908
Floods	-0.0276483	0.0134734	-2.05	0.047	-0.0549009	-0.0003957
Storms	-0.0025713	0.0089111	-0.29	0.774	-0.0205958	0.0154531
Neonatal mortality rate	0.0734174	0.0011754	62.46	<0.001	0.0710399	0.0757949
GNI per capita	0.0006197	0.0000721	8.59	<0.001	0.0004737	0.0007656
**Model-3**						
Extreme temperature events	-0.0640644	0.0303189	-2.11	0.041	-0.1253901	-0.0027386
Floods	-0.0494053	0.0199667	-2.47	0.018	-0.0897918	-0.0090189
Storms	-0.0078053	0.013237	-0.59	0.559	-0.0345797	0.0189691
Infant mortality rate	0.0436789	0.001048	41.68	<0.001	0.0415592	0.0457986
GNI per capita	0.0003886	0.0001031	3.77	0.001	0.0001801	0.000597
**Model-4**						
Extreme temperature events	-0.0685981	0.0315871	-2.17	0.036	-0.1324891	-0.0047072
Floods	-0.0519952	0.020819	-2.5	0.017	-0.0941056	-0.0098848
Storms	-0.008896	0.0138015	-0.64	0.523	-0.0368122	0.0190201
Male infant mortality rate	0.0407757	0.0010217	39.91	<0.001	0.0387092	0.0428422
GNI per capita	0.0003833	0.0001075	3.57	0.001	0.0001659	0.0006007
**Model-5**						
Extreme temperature events	0.049556	0.0545905	0.91	0.373	-0.0628752	0.1619872
Floods	0.0084307	0.0339281	0.25	0.806	-0.0614455	0.0783069
Storms	0.0674067	0.025088	2.69	0.013	0.0157369	0.1190765
Contraceptive prevalence rate	-0.0854929	0.0043022	-19.87	<0.001	-0.0943536	-0.0766323
GNI per capita	-0.0001902	0.0002197	-0.87	0.395	-0.0006427	0.0002623

Table 7 ARIMA Regression on total fertility rate by the total extreme climate events and other characteristics

**Variables**	**β coefficient**	**Std. Err.**	**t**	**p-value**	**95% CI**	
**Model-1**						
Total extreme climate events	-0.0000789	0.0000366	-2.15	0.031	-0.00015	-7050000
Under five (U5) mortality rate both sex	0.004864	0.002641	1.84	0.066	-0.00031	0.010041
**Model-2**						
Total extreme climate events	-0.0000553	0.0000327	-1.69	0.091	-0.00012	0.00000892
Neonatal mortality rate	0.003483	0.001964	1.77	0.076	-0.00037	0.007332

**Model-3**						
Total extreme climate events	-0.0000805	0.0000313	-2.57	0.01	-0.00014	-0.0000192
Infant mortality rate	-0.00226	0.002329	-0.97	0.331	-0.00683	0.0023
**Model-4**						
Total extreme climate events	-0.0000891	0.0000353	-2.52	0.012	-0.00016	-0.0000199
Male infant mortality rate	-0.00044	0.002087	-0.21	0.833	-0.00453	0.00365
**Model-5**						
Total extreme climate events	-0.0000565	0.000138	-0.41	0.683	-0.00033	0.000215
Contraceptive prevalence rate	-0.00018	0.000202	-0.87	0.382	-0.00057	0.000219

Figures

Figure 1 Extreme climate event records (Total) for Bangladesh, 1960–2018

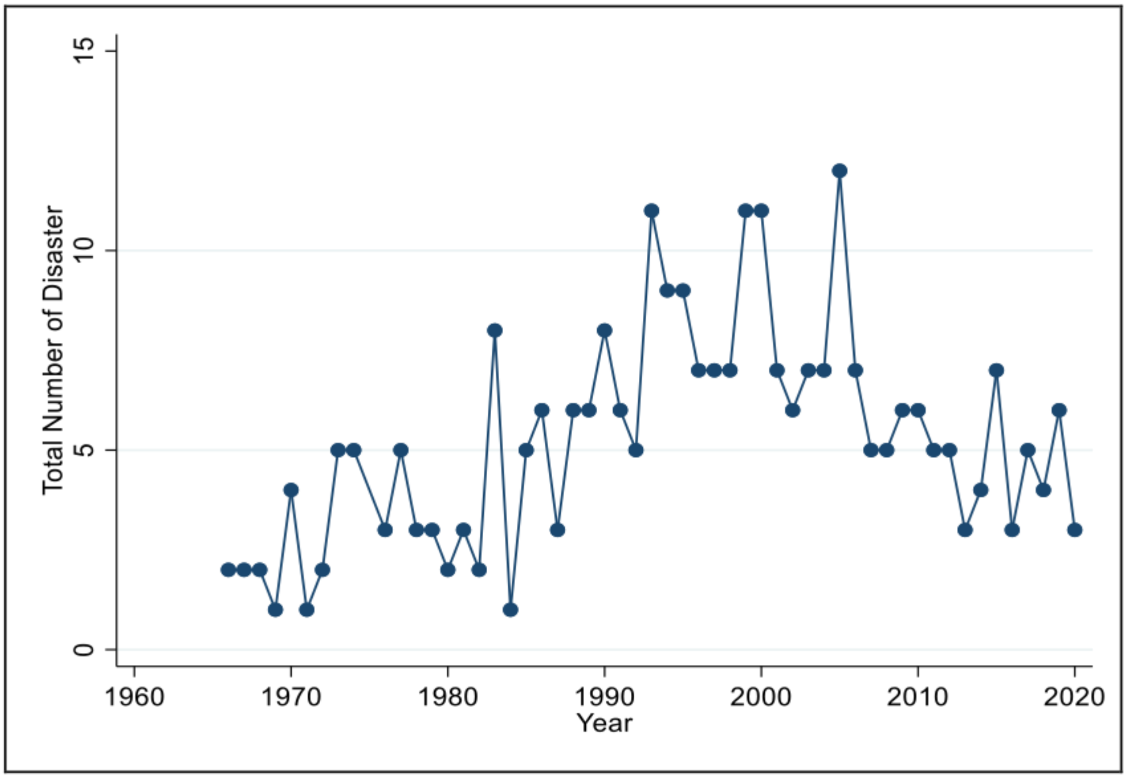

Figure 2 Trends of TFR and CPR for Bangladesh, 1960–2018

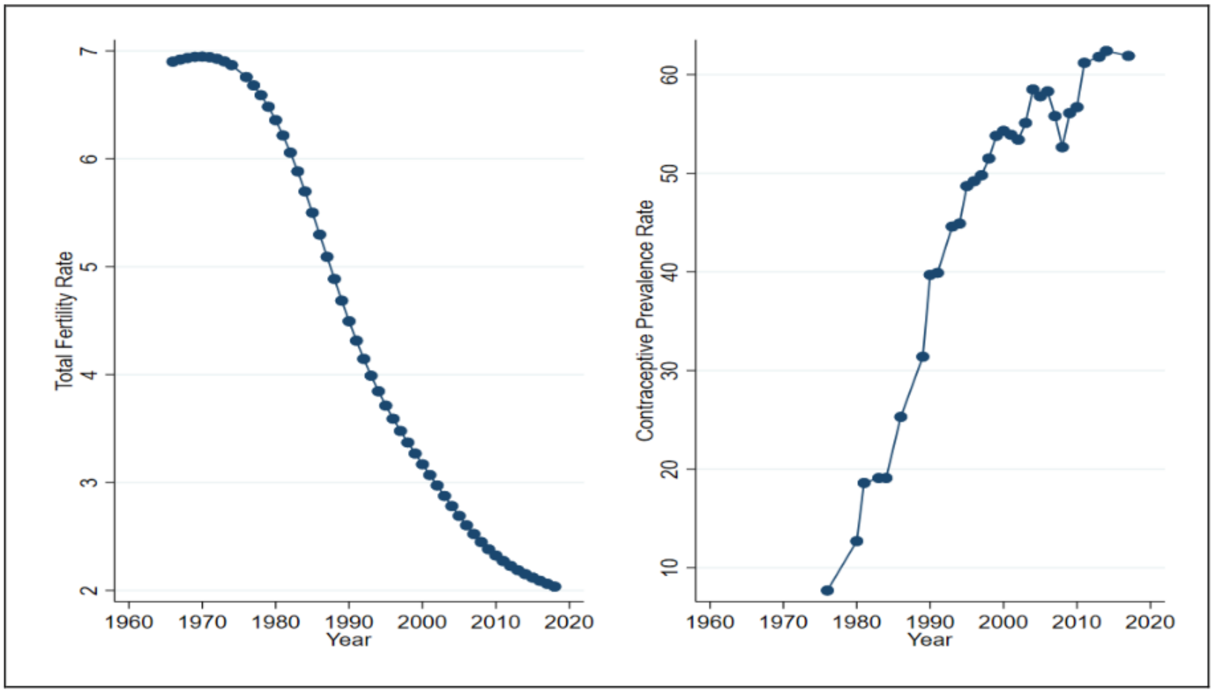

Note: The unit of all variables is annual.

Figure 3 Trends of Neonatal, infant, male infant and under-five mortality rate for Bangladesh, 1960–2018

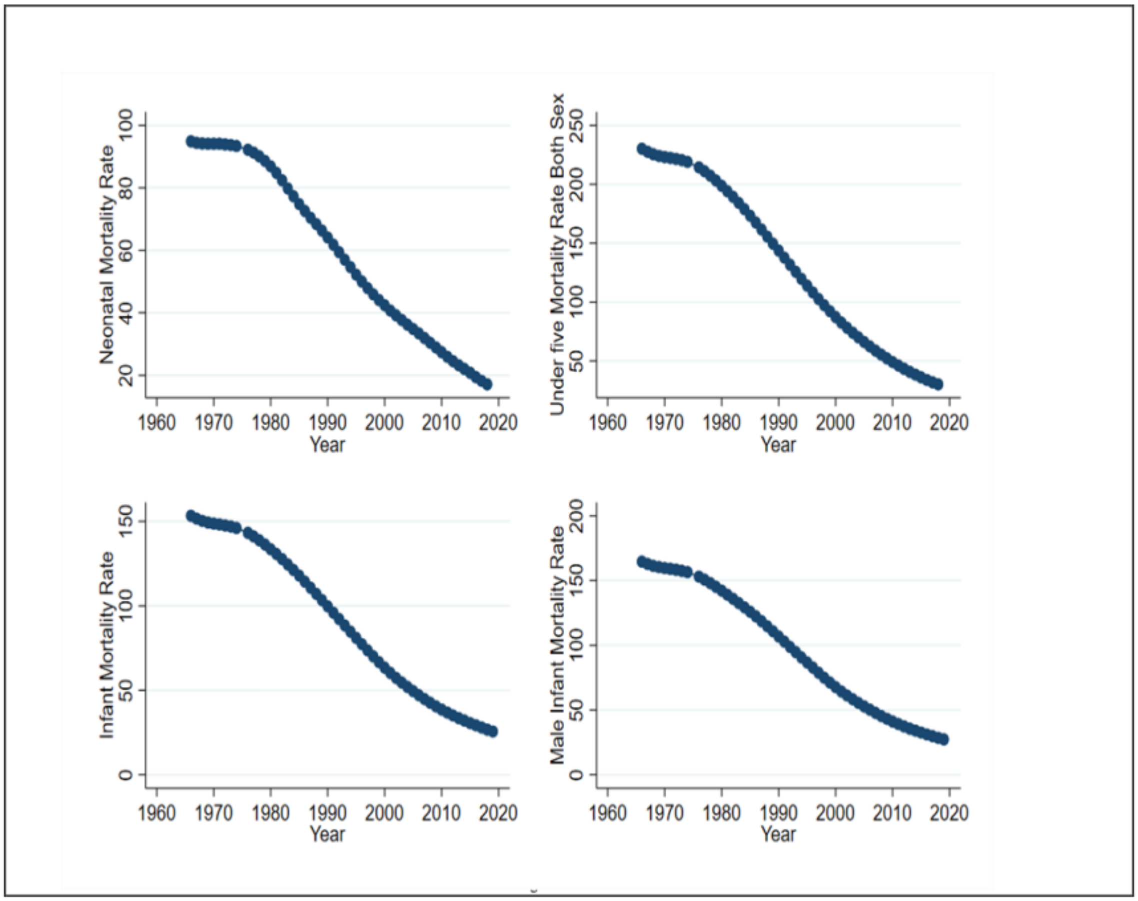

Note: The unit of all variables is annual.

Figure 4 GNI per capita (Atlas method, US$) for Bangladesh, 1960–2018

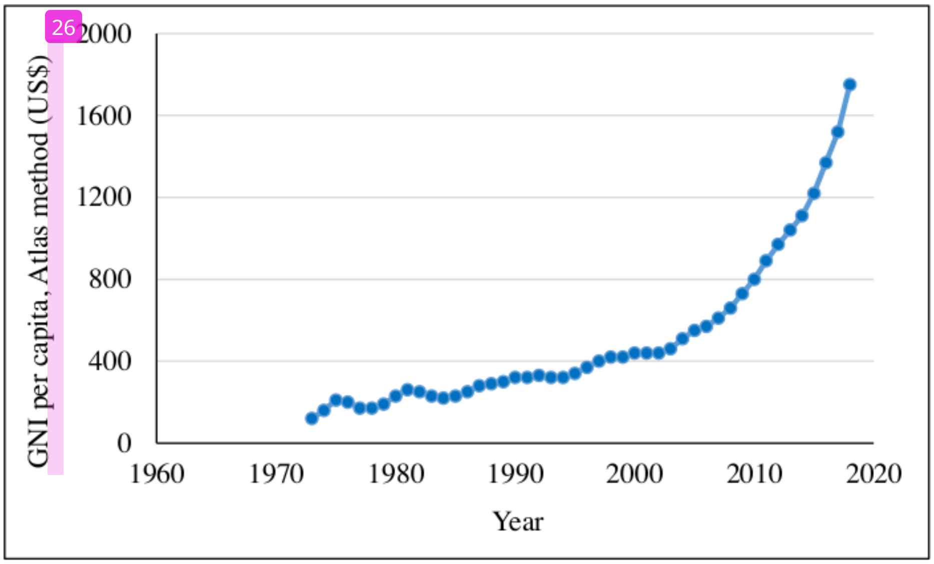

References

[1] Jiang, L., & Hardee, K. (2011). How do recent population trends matter to climate change? Population Research and Policy Review, 30(2), 287-312.

[2] Casey, G., Shayegh, S., Moreno–Cruz, J., Bunzl, M., Galor, O., & Caldeira, K. (2019). The impact of climate change on fertility. Environmental Research Letters, 14(5).

[3] Muttarak, R. (2021). Demographic perspectives in research on global environmental change. Population Studies, 75, 77-104.

[4] Caldwell, J., Barkat-e-Khuda, Caldwell, B., Pieris, I., & Caldwell, P. (1999). The Bangladesh Fertility Decline: An Interpretation. Population and Development Review, 25(1), 67-84.

[5] Cleland, J. C., Phillips, J. F., Amin, S., & Kamal, G. M. (1994). The determinants of reproductive change in Bangladesh—success in a challenging environment. World Bank Regional and Sectoral Studies. Washington, DC: The World Bank.

[6] Haq, S. M. A. (2018). Underlying causes and the impacts of disaster events (floods) on fertility decision in rural Bangladesh. Environmental and Socio-economic Studies, 6(3), 24–35.

[7] Ahmed, K. J., Haq, S. M. A., & Bartiaux, F. (2019). The nexus between extreme weather events, sexual violence, and early marriage: a study of vulnerable populations in Bangladesh. Population and Environment, 40(3), 303-324.

[8] Miyaji, M., Okazaki, K., & Ochiai, C. (2020). A study on the use of cyclone shelters in Bangladesh. Japan Architectural Review, 3, 590–600.

[9] Haq, S. M. A., & Ahmed, K. J. (2019). Is fertility preference related to perception of the risk of child mortality, changes in landholding, and type of family? A comparative study on populations vulnerable and not vulnerable to extreme weather events in Bangladesh. Population Review, 58(2), 61-99.

[10] Hossain, M., Mani, K. K. C., Sidik, S. M., Hayati, K. S., & Rahman, A. K. M. F. (2015). Socio-demographic, environmental and caring risk factors for childhood drowning deaths in Bangladesh. BMC Pediatrics, 15(1), 114.

[11] Hamamatsu, Y., Inoue, Y., Watanabe, C., & Umezaki, M. (2014). Impact of the 2011 earthquake on marriages, births and the secondary sex ratio in Japan. Journal of Biosocial Science, 46(6), 830-841.

[12] Alam, M. A., Chamroonsawasdi, K., Chansatitporn, N., & Munsawaengsub, C. S. (2018). Regional variations of fertility control behavior among rural reproductive women in Bangladesh: A hierarchical analysis. Behavioral Sciences, 8(8), 68.

[13] Kabir, A., Ali, R., Islam, M. S., Kawsar, L. A., & Islam, M. A. (2009). A comparison of regional variations of fertility in Bangladesh. International Quarterly of Community Health Education, 29(3), 275-291.

[14] Sellers, S., & Gray, C. (2019). Climate shocks constrain human fertility in Indonesia. World Development, 117, 357-369.

[15] Saha, U. R., & Bairagi, R. (2007). Inconsistencies in the relationship between contraceptive use and fertility in Bangladesh. International Family Planning Perspectives, 33(1), 31-37.

[16] Hasan, M., & Sabiruzzaman. (2008). Factors affecting fertility behavior in Bangladesh: A probabilistic approach. Research Journal of Applied Sciences, 3(1), 70-76.

[17] Gray, C., & Mueller, V. (2012). Natural disasters and population mobility in Bangladesh. Proceedings of the National Academy of Sciences, 109(16), 6000-6005.

[18] Carta, G., Dalfonso, A., Colagrande, I., Catana, P., Casacchia, M., & Patacchiola, F. (2012). Post-earthquake birth-rate evaluation using the brief cope. Journal of Maternal-Fetal and Neonatal Medicine, 25(11), 2411-2414.

[19] Akmam, W. (2002). Women’s education and fertility rates in developing countries, with special reference to Bangladesh. Eubios Journal of Asian and International Bioethics, 12(4), 138-142.

[20] Bairagi, R., & Datta, A. K. (2001). Demographic transition in Bangladesh: What happened in the twentieth century and what will happen next? Asia Pacific Population Journal, 16(4), 3-16.

[21] Bairagi, R. (2001). Effects of sex preference on contraceptive use, abortion, and fertility in Matlab, Bangladesh. International Family Planning Perspectives, 27(3), 137-143.

[22] Hossain, F., & Karim, R. (2013). Determination of total fertility rate of Bangladesh using Bongaarts model. Journal of Biometrics and Biostatistics, 4(5).

[23] Ahmed, S., Nahar, S., Amin, N., & Shirin, S. (2009). Age at marriage and fertility pattern of adolescent married women in rural Bangladesh. IMC Journal of Medical Science, 1(2), 9-12.

[24] Islam, A. Z., Mondal, M. N. I., Khatun, M. L., Rahman, M. M., Islam, M. R., Mostofa, M. G., & Hoque, M. N. (2016). Prevalence and determinants of contraceptive use among employed and unemployed women in Bangladesh. International Journal of MCH and AIDS, 5(2), 92.
[25] Rabbi, A. M. F. (2012). Mass media exposure and its impact on fertility: Current scenario of Bangladesh. Journal of Scientific Research, 4(2).
[26] Streatfield, P. K., & Kamal, N. (2013). Population and family planning in Bangladesh. The Journal of the Pakistan Medical Association, 63(4 Suppl 3), S73–S81.
[27] Schuler, S. R., Hashemi, S. M., & Jenkins, A. H. (1995). Bangladesh's family planning success story: A gender perspective. International Family Planning Perspectives, 21(132).
[28] National Institute of Population Research and Training (NIPORT), International Centre for Diarrhoeal Disease Research, Bangladesh (ICDDRB), & MEASURE Evaluation. (2019). Bangladesh district level socio–demographic and health care utilization indicators. Dhaka, Bangladesh, and Chapel Hill, NC, USA: NIPORT, ICDDRB, and MEASURE Evaluation.
[29] Cohan, C. L., & Cole, S. W. (2002). Life course transitions and natural disaster: Marriage, birth, and divorce following Hurricane Hugo. Journal of Family Psychology, 16(1), 14-25.
[30] Davis, J. (2017). Fertility after natural disaster: Hurricane Mitch in Nicaragua. Population and Environment, 38(4), 448-464.
[31] Antipova, A., & Curtis, A. (2015). The post-disaster negative health legacy: pregnancy outcomes in Louisiana after Hurricane Andrew. Disasters, 39(4), 665-686.
[32] Evans, R. W., Hu, Y. Y., & Zhao, Z. (2010). The fertility effect of catastrophe: US hurricane births. Journal of Population Economics, 23(1), 1-36.
[33] Haq, S. M. A. (2013). Nexus between perception, environment and fertility: A study on indigenous people in Bangladesh. Sustainable Development, 21(6), 372–384.
[34] Haq, S. M. A. (2023). The impact of extreme weather events on fertility preference and gender preference in Bangladesh. Frontiers in Environmental Science, 11, 1095460.
[35] Grace, K. (2017). Considering climate in studies of fertility and reproductive health in poor countries. Nature Climate Change, 7(7), 479.
[36] Hogan, D. J., & Marandola, J. E. (2012). Bringing a population-environment perspective to hazards research. Population and Environment, 34, 3-21.
[37] Kabir, A., Jahan, R., Islam, M. S., & Ali, R. (2001). The effect of child mortality on fertility. Journal of Medical Sciences, 1(6), 377-380.
[38] Sandberg, J. (2006). Infant mortality, social networks, and subsequent fertility. American Sociological Review, 71(2), 288-309.
[39] Datar, A., Liu, J., Linnemayr, S., & Stecher, C. (2012). The impact of natural disasters on child health and investments in rural India. Social Science and Medicine, 76, 83-91.
[40] Nobles, J., Frankenberg, E., & Thomas, D. (2015). The effects of mortality on fertility: Population dynamics after a natural disaster. Demography, 52(1), 15-38.
[41] Chen, M., Haq, S. M. A., Ahmed, K. J., Hussain, A. B., & Ahmed, M. N. (2021). The link between climate change, food security and fertility: The case of Bangladesh. PLoS One, 16(10), e0258196.

[42] Simon, D. H. (2017). Exploring the influence of precipitation on fertility timing in rural Mexico. Population and Environment, 38(4), 407-423.

[43] Carrico, A. R., & Donato, K. (2019). Extreme weather and migration: evidence from Bangladesh. Population and Environment, 41, 1–31.

[44] Seltzer, N., & Nobles, J. (2017). Post-disaster fertility: Hurricane Katrina and the changing racial composition of New Orleans. Population and Environment, 38(4), 465–490.

[45] Barreca, A. (2017). Does hot weather affect human fertility? IZA World of Labor, 375.

[46] Barreca, A., Deschenes, O., & Guldi, M. (2018). Maybe next month? Temperature shocks and dynamic adjustments in birth rates. Demography, 55(4), 1269–1293.

[47] Cho, H. (2020). Ambient temperature, birth rate, and birth outcomes: evidence from South Korea. Population and Environment, 41(3), 330–346.

[48] Tong, V., Zotti, M., & Hsia, J. (2011). Impact of the Red River catastrophic flood on women giving birth in North Dakota, 1994-2000. Maternal and Child Health Journal, 15(3), 281-288.

[49] Harville, E. W., Xiong, X., David, M., & Buekens, P. (2020). The paradoxical effects of Hurricane Katrina on births and adverse birth outcomes. American Journal of Public Health, 110(10), 1466-1471.

[50] Norling, J. (2022). Fertility following natural disasters and epidemics in Africa. The World Bank Economic Review, 36(4), 955-971.

[51] Lam, D., & Miron, J. (1996). The effects of temperature on human fertility. Demography, 33(3), 291-305.

[52] Helle, S., Helama, S., & Jokela, J. (2008). Temperature-related birth sex ratio bias in historical Sami: Warm years bring more sons. Biology Letters, 4(1), 60.

[53] Thiede, B. C., Ronnkvist, S., Armao, A., et al. (2022). Climate anomalies and birth rates in sub-Saharan Africa. Climatic Change, 171, 5.

[54] Philibert, A., Tourigny, C., Coulibaly, A., & Fournier, P. (2013). Birth seasonality as a response to a changing rural environment (Kayes region, Mali). Journal of Biosocial Science, 45(4), 547–565.

[55] Cain, M. (1981). Risk and insurance: perspectives on fertility and agrarian change in India and Bangladesh. Population and Development Review, 7(3), 435-474.

[56] Finlay, E. J. (2009). Fertility response to natural disasters: The case of three high mortality earthquakes. World Bank Policy Research Working Paper, 4883. Available at http://papers.ssrn.com/sol3/papers.cfm?abstract_id=1372960

[57] Pörtner, C. C. (2008). Gone with the wind? Hurricane risk, fertility and education. Working papers, University of Washington, Washington.

[58] Qin, L., Luo, S., Li, X., Wang, Y., & Li, S. (2009). Fertility assistance program following the Sichuan earthquake in China. International Journal of Gynecology and Obstetrics, 104(3), 182-183.

[59] Agadjanian, V., & Prata, N. (2002). War, peace, and fertility in Angola. Demography, 39(2), 215-231.

[60] Frankenberg, E., Laurito, M. M., & Thomas, D. (2015). Demographic impact of disasters. In J. D. Wright (Ed.), International Encyclopedia of the Social and Behavioral Sciences (2nd ed., pp. 101-108). Elsevier.

[61] BMD. (2020). Module 2: Climatology. Retrieved from https://www.bmd.gov.bd/file/2021/02/16/pdf/109889.pdf

[62] Bongaarts, J. (2017). The effect of contraception on fertility: Is sub-Saharan Africa different? Demographic Research, 37(6), 129-146.

[63] Stover, J., & Winfrey, W. (2017). The effects of family planning and other factors on fertility, abortion, miscarriage, and stillbirths in the Spectrum model. BMC Public Health, 17(4), 43-50.

[64] Vollset, S. E., Goren, E., Yuan, C. W., Cao, J., Smith, A. E., Hsiao, T., ... & Bisignano, C. (2020). Fertility, mortality, migration, and population scenarios for 195 countries and territories from 2017 to 2100: A forecasting analysis for the Global Burden of Disease Study. The Lancet, 396(10258), 1285-306.

[65] Ahmed, K. J., & Tan, Y. (2021). Assessing and mapping spatial variations in climate change and climatic hazards in Bangladesh. In G.M.M. Alam, M.O. Erdiaw-Kwasie, G.J. Nagy, & W. Leal Filho (Eds.), Climate vulnerability and resilience in the Global South: Human adaptations for sustainable futures (pp. 465-486).

[66] Uddin, M. A., Ichihashi, M., & Barua, S. (2022). Financial Sector Development and the Preference for Informal Remittance Channels: Evidence from Bangladesh. The Journal of Development Studies, 14, 1-27.

[67] Bureau of Manpower, Employment and Training (BMET). (2022, July). Reterived August 10, 2022, from http://www.old.bmet.gov.bd/BMET/viewStatReport.action?reportnumber=24.

[68] Ahinkorah, B. O., Seidu, A. A., Armah-Ansah, E. K., Ameyaw, E. K., Budu, E., & Yaya, S. (2021). Socio-economic and demographic factors associated with fertility preferences among women of reproductive age in Ghana: Evidence from the 2014 demographic and health survey. Reproductive Health, 18, 2.

[69] Lin, C. Y. C. (2010). Instability, investment, disasters, and demography: Natural disasters and fertility in Italy (1820–1962) and Japan (1671–1965). Population and Environment, 31, 255–281.

[70] Blanc, A. K. (2004). The role of conflict in the rapid fertility decline in Eritrea and prospects for the future. Studies in Family Planning, 35(4), 236-245.

[71] Clifford, D., Falkingham, J., & Hinde, A. (2010). Through civil war, food crisis and drought: Trends in fertility and nuptiality in Post-Soviet Tajikistan. European Journal of Population, 26(3), 325-350.

[72] Kissinger, P., Schmidt, N., Sanders, C., & Liddon, N. (2007). The effect of the hurricane Katrina disaster on sexual behavior and access to reproductive care for young women in New Orleans. Sexually Transmitted Diseases, 34(11), 883–886.

[73] Leyser–Whalen, O., Rahman, M., & Berenson, A. B. (2011). Natural and social disasters: Racial inequality in access to contraceptives after Hurricane Ike. Journal of Women's Health, 20(12), 1861–1866.

[74] National Institute of Population Research and Training (NIPORT), & ICF. (2020). Bangladesh Demographic and Health Survey 2017-18. Dhaka, Bangladesh, and Rockville, Maryland, USA: NIPORT and ICF.

[75] Islam, S., Islam, M. A., & Padmadas, S. S. (2010). High fertility regions in Bangladesh: A marriage cohort analysis. Journal of Biosocial Science, 42(6), 705-719.

[76] Ahmed, K. J., Tan, Y., & Rudd, D. (2023). Changing fertility, child mortality and contraceptive prevalence rates in Bangladesh: Effects of disaster risk reduction and family planning programs. In W. Leal Filho, D. G. Vidal, & M. A. P. Dinis (Eds.), Climate change and health hazards: Addressing hazards to human and environmental health from a changing climate (pp. 381-406). Springer Nature Switzerland.

[77] EM-DAT. The International Disaster Database. (2018). Available from https://www.emdat.be/

Clean Version_HELIYON_31.May.2024.doc

ORIGINALITY REPORT

14%
SIMILARITY INDEX

PRIMARY SOURCES

1	www.researchsquare.com<br>Internet	571 words — 5%
2	hdl.handle.net<br>Internet	117 words — 1%
3	"Climate Vulnerability and Resilience in the Global South", Springer Science and Business Media LLC, 2021<br>Crossref	54 words — < 1%
4	assets.researchsquare.com<br>Internet	53 words — < 1%
5	www.nature.com<br>Internet	46 words — < 1%
6	bmcpublichealth.biomedcentral.com<br>Internet	39 words — < 1%
7	Khandaker Jafor Ahmed, Yan Tan, Dianne Rudd. "Exploring the relationship between changes in fertility and disasters: a review of the literature", Journal of Population Research, 2023<br>Crossref	37 words — < 1%
8	openaccess.hacettepe.edu.tr<br>Internet	34 words — < 1%

9	link.springer.com	Internet	30 words	— < 1%
10	www.data4impactproject.org	Internet	27 words	— < 1%
11	Otavio T. Ranzani, Anjani Kalra, Chiara Di Girolamo, Ariadna Curto et al. "Urban-rural differences in hypertension prevalence in low-income and middle-income countries, 1990–2020: A systematic review and meta-analysis", PLOS Medicine, 2022	Crossref	24 words	— < 1%
12	thesis.eur.nl	Internet	24 words	— < 1%
13	Shah Md Atiqul Haq. "The impact of extreme weather events on fertility preference and gender preference in Bangladesh", Frontiers in Environmental Science, 2023	Crossref	19 words	— < 1%
14	www.researchgate.net	Internet	18 words	— < 1%
15	jscholarship.library.jhu.edu	Internet	17 words	— < 1%
16	www.frontiersin.org	Internet	17 words	— < 1%
17	www.igi-global.com	Internet	17 words	— < 1%
18	img1.wsimg.com	Internet	16 words	— < 1%

19	web.archive.org<br>Internet	16 words — < 1%
20	www2.mdpi.com<br>Internet	15 words — < 1%
21	hqlo.biomedcentral.com<br>Internet	14 words — < 1%
22	dataaspirant.com<br>Internet	13 words — < 1%
23	helda.helsinki.fi<br>Internet	13 words — < 1%
24	Indrani Barui, Shabari Bhakta, Kapil Ghosh, Rajib Shaw. "Assessment of Household Vulnerability to Embankment Breaching in the Coastal Area of the Indian Sundarban", International Journal of Disaster Risk Reduction, 2024<br>Crossref	12 words — < 1%
25	perpustakaan.poltekkes-malang.ac.id<br>Internet	12 words — < 1%
26	unsworks.unsw.edu.au<br>Internet	12 words — < 1%
27	Hyunkuk Cho. "Ambient temperature, birth rate, and birth outcomes: evidence from South Korea", Population and Environment, 2019<br>Crossref	11 words — < 1%
28	files.eric.ed.gov<br>Internet	11 words — < 1%
29	academicjournals.org	

Internet

10 words — < 1%

30 ebin.pub
Internet

10 words — < 1%

31 pure.iiasa.ac.at
Internet

10 words — < 1%

32 repository.charlotte.edu
Internet

10 words — < 1%

33 www.jdsupra.com
Internet

10 words — < 1%

34 www.springerprofessional.de
Internet

10 words — < 1%

35 www.ulster.ac.uk
Internet

10 words — < 1%

36 Isabel H. McLoughlin Brooks. "Gender, climate and landowning: Sources of variability in the weather pattern change and ideal fertility relationship in Sahelian West Africa", Vienna Yearbook of Population Research, 2024
Crossref

9 words — < 1%

37 Ronald R. Rindfuss, Martin Piotrowski, Varachai Thongthai, Pramote Prasartkul. "Measuring housing quality in the absence of a monetized real estate market", Population Studies, 2007
Crossref

9 words — < 1%

38 etheses.lse.ac.uk
Internet

9 words — < 1%

39 ugspace.ug.edu.gh:8080
Internet

9 words — < 1%

40 www.ncbi.nlm.nih.gov
Internet
9 words — < 1%

41 "Climate Change Strategies: Handling the Challenges of Adapting to a Changing Climate", Springer Science and Business Media LLC, 2023
Crossref
8 words — < 1%

42 Hassan Eini-Zinab. "Multidimensional life-table analysis of the effect of child mortality on total fertility in India, 1992–93, 1998–99, 2005–06", Population Studies, 2013
Crossref
8 words — < 1%

43 JONG-WHA LEE. "DETERMINANTS OF FERTILITY IN THE LONG RUN", The Singapore Economic Review, 2019
Crossref
8 words — < 1%

44 John Bongaarts, Dennis Hodgson. "Fertility Transition in the Developing World", Springer Science and Business Media LLC, 2022
Crossref
8 words — < 1%

45 Khan, Nizamuddin. "Spousal Perspectives and Attitudes Towards Reproductive Behaviour: A Study of Young Married Couples in Ratlam District of Madhya Pradesh.", International Institute for Population Sciences University, 2021
ProQuest
8 words — < 1%

46 Md. Azad Uddin, Masaru Ichihashi, Shubhasish Barua. "Financial Sector Development and the
8 words — < 1%

Preference for Informal Remittance Channels: Evidence from Bangladesh", The Journal of Development Studies, 2022
Crossref

47 Olivier Deschênes, Enrico Moretti. "Extreme Weather Events, Mortality, and Migration", Review of Economics and Statistics, 2009 — 8 words — < 1%
Crossref

48 Samuel Sellers, Clark Gray. "Climate shocks constrain human fertility in Indonesia", World Development, 2019 — 8 words — < 1%
Crossref

49 Shah Md. Atiqul Haq, Khandaker Jafor Ahmed. "Does the perception of climate change vary with the socio-demographic dimensions? A study on vulnerable populations in Bangladesh", Natural Hazards, 2016 — 8 words — < 1%
Crossref

50 dash.harvard.edu — 8 words — < 1%
Internet

51 digital.library.adelaide.edu.au — 8 words — < 1%
Internet

52 discovery.researcher.life — 8 words — < 1%
Internet

53 dodl.klyuniv.ac.in — 8 words — < 1%
Internet

54 economics.brown.edu — 8 words — < 1%
Internet

55 eprints.soton.ac.uk — 8 words — < 1%
Internet

56	pdfcookie.com<br>Internet	8 words — < 1%
57	psasir.upm.edu.my<br>Internet	8 words — < 1%
58	pure-oai.bham.ac.uk<br>Internet	8 words — < 1%
59	www.isoss.net<br>Internet	8 words — < 1%
60	"Africa's Population: In Search of a Demographic Dividend", Springer Science and Business Media LLC, 2017<br>Crossref	7 words — < 1%
61	"International Handbook of Population and Environment", Springer Science and Business Media LLC, 2022<br>Crossref	7 words — < 1%
62	eprints.kingston.ac.uk<br>Internet	7 words — < 1%
63	Global Migration Issues, 2014.<br>Crossref	6 words — < 1%
64	Khandaker Jafor Ahmed, Shah Md Atiqul Haq. "Perceived risk of child mortality and fertility choices in climate-vulnerable regions of Bangladesh", Humanities and Social Sciences Communications, 2024<br>Crossref	6 words — < 1%

EXCLUDE QUOTES	OFF	EXCLUDE SOURCES	OFF
EXCLUDE BIBLIOGRAPHY	ON	EXCLUDE MATCHES	OFF
